# The impact of generative AI health knowledge acquisition on adolescents’ physical activity: the mediating role of exercise self-efficacy and the moderating effect of health information literacy

**DOI:** 10.3389/fpubh.2025.1705265

**Published:** 2026-01-08

**Authors:** Qunqun Sun, Hao Gou, Luyao Xiang, Chang Hu, Yuan Fang

**Affiliations:** 1 College of Physical Education, Qiannan Normal University for Nationalities, Duyun, China; 2 Zhuhai Campus, Zunyi Medical University, Zhuhai, China; 3 College of Physical Education, Jiangxi Normal University, Nanchang, China

**Keywords:** generative AI health knowledge acquisition, physical activity, exercise self-efficacy, health information literacy, adolescents

## Abstract

**Background:**

This study investigates the impact of generative artificial intelligence health knowledge acquisition (GAI-HKA) on adolescents’ physical activity (PA), with a focus on the mediating role of exercise self-efficacy (ESE) and the moderating role of health information literacy (HIL).

**Methods:**

Using stratified cluster random sampling, a total of 2,306 valid questionnaires were collected from 10 secondary schools across five cities in southern China. Data were obtained using the self-developed Generative AI Health Knowledge Acquisition Scale, the Adolescent Exercise Self-Efficacy Scale, the Health Information Literacy Scale, and Liang’s revised Physical Activity Rating Scale. Structural equation modelling with PROCESS v3.5 macro (5,000 bootstraps) was employed to test direct, mediating, and moderating effects.

**Results:**

GAI-HKA was significantly and positively associated with adolescents’ PA (*β* = 0.376, *p* < 0.001). ESE partially mediated this relationship (indirect effect = 0.065, 95% CI [0.052, 0.079]). HIL positively moderated the pathway from GAI-HKA to ESE (interaction term *β* = 0.079, *p* < 0.001); the effect was more substantial among adolescents with high HIL (*β* = 0.367) compared with those with low HIL (*β* = 0.209).

**Conclusion:**

GAI-HKA directly enhances adolescents’ PA and indirectly promotes exercise behavior through strengthening ESE. Moreover, higher HIL amplifies the positive effect of AI-based health knowledge on ESE. These findings suggest that schools and families should integrate efforts to cultivate adolescents’ information literacy and efficacy beliefs when promoting AI-based health interventions, thereby maximizing the benefits of digital technologies in fostering youth physical activity.

## 1 Introduction

In recent years, breakthroughs in generative artificial intelligence (GAI) across multimodal content generation—text, images, and speech—have positioned it as one of the most disruptive information acquisition tools since the advent of search engines (1–3). In the health domain, models such as ChatGPT, DeepSeek, and ERNIE Bot can instantly respond to complex queries such as how to build muscle scientifically or how to relieve exam anxiety (4, 5), providing the public with low-cost, personalized, and round-the-clock health knowledge services (6, 7). Adolescents, in particular, might use GAI to request personalized workout routines tailored to their fitness levels, receive dietary advice specific to certain sports, or understand the benefits of specific exercises (e.g., What are the best exercises for improving basketball shooting accuracy?) (8). Recent large-scale surveys indicate that generative AI has already become embedded in adolescents’ everyday digital routines: for example, national data from the United Kingdom and the United States suggest that roughly one third to three quarters of 13–18-year-olds have used generative AI tools at least once (9). Around 40% of teenagers report using them to support schoolwork or homework (10, 11). Beyond academic assistance, adolescents most commonly employ generative AI for explaining or summarizing learning materials, creative content production, language translation, and entertainment, and an increasing proportion report using these tools to seek information related to physical and mental health, diet, and exercise that they may be reluctant to discuss with adults (12). Emerging reports from China similarly point to widespread exposure to AI-enabled products among minors, particularly in urban areas where schools are beginning to encourage structured experimentation with generative AI in educational settings (13).

However, most existing research has primarily focused on adults or patients with chronic conditions (14–16). At the same time, relatively little attention has been given to adolescents, who are at a critical stage for shaping lifelong health behaviors (17). Adolescents generally exhibit insufficient physical activity (PA), but they show a remarkable enthusiasm for adopting new technologies (18, 19). Whether and how generative AI health knowledge acquisition (GAI-HKA) influences their exercise behaviors remains unexplored (20, 21). Against this backdrop, the present study investigates Chinese adolescents to examine the potential promoting or inhibiting effects of GAI-HKA on PA, aiming to provide scientific evidence for leveraging AI technologies to effectively foster healthy behaviors among youth.

### 1.1 The relationship between generative AI health knowledge acquisition and adolescents’ physical activity

GAI-HKA refers to the process by which individuals actively use generative AI tools—such as large language models—to acquire health-related information when faced with health questions or behavioral improvement needs. This process includes not only the frequency of use, but also the types of queries asked and the perceived quality of the information obtained. It involves iterative inquiries, content comprehension, and the subsequent application of the knowledge in daily life (22). Its core feature lies in the system’s ability to provide instant, personalized, and conversational outputs that integrate health recommendations in a comprehensive manner (23–25). According to the Unified Theory of Acceptance and Use of Technology (UTAUT), the perceived usefulness and ease of use of emerging technologies significantly increase their frequency of adoption, thereby strengthening behavioral intentions (26). In the context of health, adolescents may perceive GAI as highly useful for obtaining quick, personalized exercise tips and easy to use due to its conversational interface. This enhances their likelihood of adopting GAI for health-related guidance and increases their motivation to engage in physical activity. Applied to a health context, this framework suggests that the more frequently adolescents rely on GAI-HKA for exercise-related guidance, the stronger their motivation and likelihood of engaging in PA (27, 28).

Longitudinal studies have demonstrated that adolescents who consistently seek exercise-related information through online platforms exhibit significant increases in vigorous PA during follow-up periods (29). Cross-sectional evidence further indicates that the frequency of online health information searches is positively associated with the likelihood of meeting recommended physical activity levels (30). These findings collectively suggest that digital health knowledge acquisition can translate into actual exercise behaviors (31, 32). Given its interactive and personalized characteristics, generative AI may amplify this effect by enhancing the efficiency of knowledge delivery (33). Therefore, it is reasonable to hypothesize that GAI-HKA has a potential facilitating role in promoting adolescents’ PA.

### 1.2 The potential mediating role of exercise self-efficacy in the relationship between generative AI health knowledge acquisition and adolescents’ physical activity

Exercise self-efficacy (ESE) refers to an individual’s subjective belief in their ability to consistently engage in PA under various circumstances (34). According to social cognitive theory (35), efficacy beliefs are not fixed traits but are shaped through continuous interactions with the environment (36, 37). When individuals receive specific, credible, and encouraging external information, their expectations for successfully performing target behaviors are strengthened (38–40). GAI-HKA provides precisely such reinforcement mechanisms—characterized by instant feedback, personalized recommendations, and persuasive positive language (28). Through each query, follow-up, and application of advice, AI systems repeatedly function as both a “virtual role model” and an “on-demand coach,” gradually cultivating adolescents’ confidence in their ability to act (I can do it) (41). Recent longitudinal evidence supports this mechanism. A follow-up study involving secondary school students revealed that those who frequently accessed online health resources and adopted exercise-related advice exhibited significantly higher levels of ESE compared to their baseline, with particularly pronounced improvements among students with initially low ESE (42).

Furthermore, self-efficacy theory posits that efficacy beliefs are a core determinant of behavior initiation, persistence, and recurrence (43). The stronger an individual’s confidence in their own abilities, the more likely they are to set ambitious goals, invest sustained effort, and persist in the face of obstacles (44–46). Recent adolescent-focused research confirmed that for every one standard deviation increase in ESE, the likelihood of meeting the daily guidelines for moderate-to-vigorous PA rose by approximately 30%, with consistent effects across gender and grade levels (47). Taken together, these two lines of evidence suggest that GAI-HKA may first enhance adolescents’ ESE, which in turn indirectly promotes sustained increases in PA.

### 1.3 The potential moderating role of health information literacy in the relationship between generative AI health knowledge acquisition and adolescents’ physical activity

Health information literacy (HIL) refers to an individual’s comprehensive ability to acquire, understand, evaluate, and apply health information to make informed health decisions (48). When HIL is high, adolescents are better able to quickly identify and filter out redundant or misleading content from generative AI outputs, integrating high-quality (49), evidence-based recommendations into actionable personal plans (50). According to the elaboration likelihood model (ELM), individuals with higher literacy levels are more likely to adopt the “central route” of information processing, engaging in more thorough cognitive elaboration (51). This process amplifies the positive effect of GAI-HKA on ESE (52), with HIL functioning as a “magnifier (53).”

In contrast, when HIL is low, adolescents may feel overwhelmed by the sheer volume and heterogeneity of AI-generated content, or even place undue trust in unverified suggestions (54, 55). This can undermine their confidence in their exercise capabilities (56). ELM suggests that individuals with lower literacy are more prone to rely on the “peripheral route,” attending to superficial cues rather than substantive arguments (51). Consequently, the positive association between GAI-HKA and ESE may be weakened—or even reversed—among those with low HIL. Therefore, it can be inferred that HIL exerts a significant moderating effect in the relationship between GAI-HKA and adolescents’ ESE: higher literacy strengthens the pathway, whereas lower literacy attenuates it.

### 1.4 Research objectives and hypotheses

In summary, this study proposes the following hypotheses:

H1: GAI-HKA positively promotes adolescents’ PA.

H2: ESE mediates the relationship between GAI-HKA and adolescents’ PA.

H3: HIL moderates the pathway from GAI-HKA to ESE.

**Figure 1:**
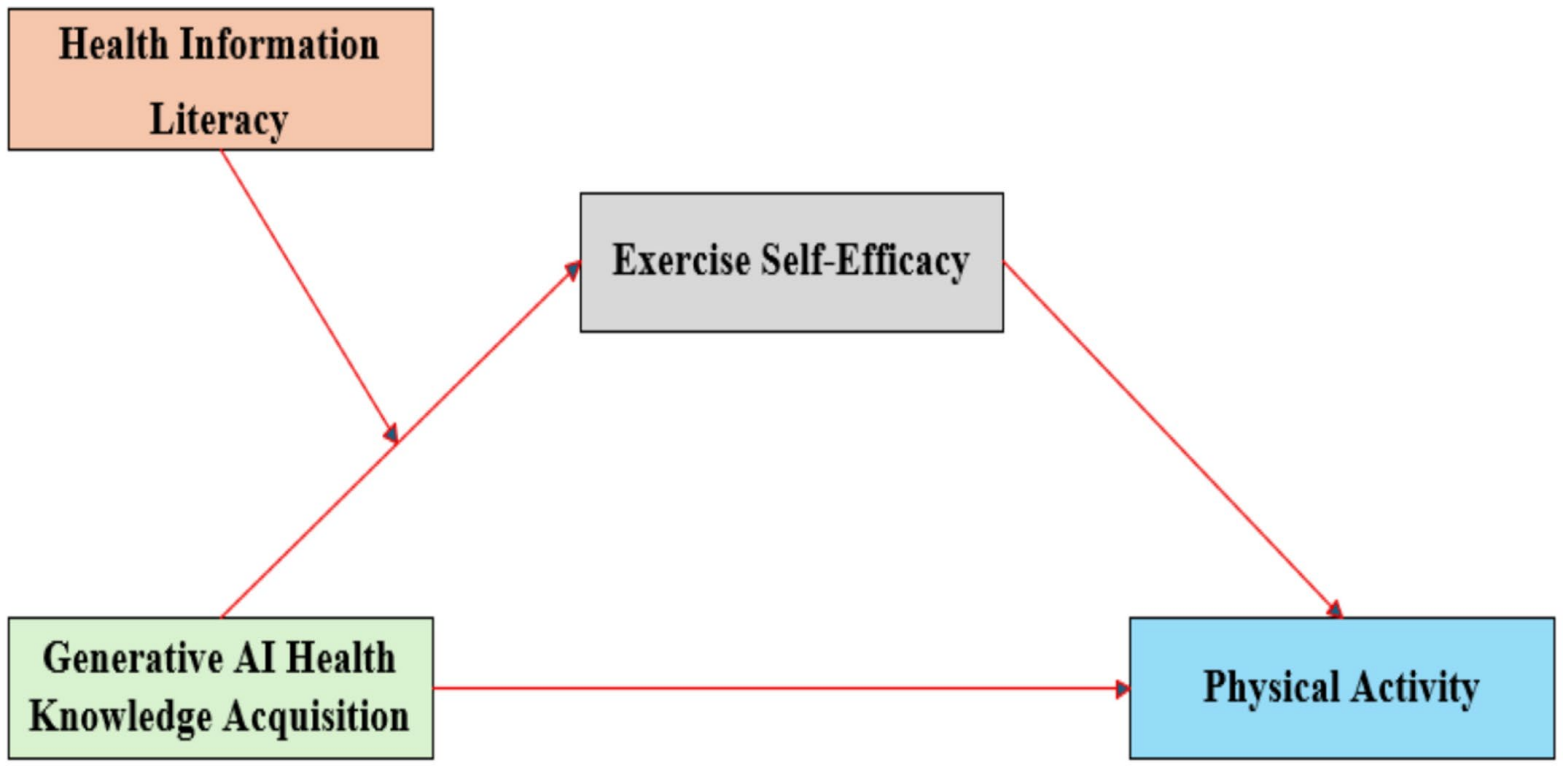
Hypothetical model.

Figure 1 illustrates the conceptual model depicting the hypothesized relationships among these variables.

## 2 Methods

### 2.1 Participants and procedures

A stratified cluster random sampling method was employed. Sample size calculation was conducted using G*Power 3.1 to determine the minimum required sample size. For the moderated mediation model employed in this study, the significance level (*α*) was set at 0.05, statistical power (1-β) at 0.80, and a medium effect size (*f*^2^ = 0.15) was assumed. The analysis indicated that with four predictors, a minimum of 150 participants would be required. Additionally, according to the empirical guideline of “10–15 participants per questionnaire item” commonly used in social science research, and given that the questionnaires used in this study contained a total of 66 items, the estimated sample size range was 660–990 participants. Based on these two methods, and to ensure the rigor and robustness of the analysis, the final minimum sample size target was set at 1,000 participants. Using a random number table, one junior high school and one senior high school were randomly selected from each of five cities in southern China, yielding a total of 10 schools. Within each school, grades were used as strata, and three classes were randomly drawn by lot from either Grade 7 and Grade 8 (junior high) or Grade 10 and Grade 11 (senior high), with each class including no fewer than 40 students. A total of 60 classes were selected (57). The field survey was conducted between March and June 2025. To ensure the feasibility of follow-up surveys, graduating classes were excluded from the study. All students in the selected classes were invited to participate, and a total of 2,580 questionnaires were distributed. After excluding invalid questionnaires (e.g., those with uniform or patterned responses, or with unreasonably short completion times), 2,306 valid responses were obtained, yielding a response rate of 89.4%.

As shown in Table 1, the participants ranged in age from 12 to 18 years (M = 14.50 ± 1.70); 45.1% were male and 54.9% were female. The grade distribution was as follows: 32.0% in Grade 7, 26.4% in Grade 8, 22.9% in Grade 10, and 18.7% in Grade 11. This study was approved by the Ethics Committee of the School of Physical Education, Qiannan Normal University for Nationalities. Written informed consent was obtained from all participants.

**Table 1:** Basic characteristics of the sample.

Variable	Sort	Frequency	Scale
Sex	Male	1,041	45.10%
	Female	1,265	54.90%
Grade	Grade 7	738	32.00%
	Grade 8	608	26.40%
	Grade 10	528	22.90%
	Grade 11	432	18.70%

### 2.2 Measuring tools

#### 2.2.1 Physical activity scale

Adolescents’ physical activity was assessed using the Physical Activity Rating Scale (PARS-3), revised by Liang (58) and Liu et al. (59). The scale evaluates exercise across three dimensions: duration, intensity, and frequency, with one item for each dimension rated on a 1–5 scale. According to the original scoring rules of PARS-3, the overall PA score is computed as PA score = intensity × frequency × (duration − 1), where each of the three items is first scored from 1 to 5 and the duration item is then transformed to (duration − 1) for calculation. Because the possible values of intensity and frequency range from 1 to 5 and the transformed duration term ranges from 0 to 4, the theoretical total score thus ranges from 0 to 100 (5 × 5 × 4), with higher scores indicating greater levels of physical activity. Consistent with Liang’s recommendations, scores ≤19 indicating low PA, scores between 20 and 42 indicating moderate PA, and scores ≥43 indicating high PA. In the present study, the Cronbach’s *α* coefficient of the scale was 0.755, demonstrating acceptable reliability.

#### 2.2.2 Exercise self-efficacy scale

Exercise self-efficacy was measured using the Adolescent Exercise Self-Efficacy Scale, developed by Yu (60). The scale consists of 24 items across six dimensions: interpersonal communication, physical health, recreational enjoyment, physical fitness, emotional experience, and life evaluation. Each item is rated on a 5-point Likert scale ranging from 1 (strongly disagree) to 5 (strongly agree), with higher scores indicating stronger exercise self-efficacy among adolescents. In the present study, the Cronbach’s *α* coefficient of the scale was 0.853, demonstrating good reliability. Confirmatory factor analysis (CFA) further indicated that the measurement model exhibited satisfactory fit indices (χ^2^/df = 1.290, RMSEA = 0.011, SRMR = 0.014, CFI = 0.996, TLI = 0.996).

#### 2.2.3 Health information literacy scale

Health information literacy was assessed using the Health Information Literacy Scale, developed by Wang et al. (61). The scale comprises 29 items across five dimensions: health information acquisition (12 items), health information evaluation (5 items), health information ethics (4 items), health information application (4 items), and health information cognition (4 items). Each item is rated on a 5-point Likert scale, ranging from 1 (very poor) to 5 (very good), with total scores ranging from 29 to 145. Higher scores reflect higher levels of HIL. In this study, the Cronbach’s *α* coefficient was 0.823, indicating good internal consistency. CFA demonstrated acceptable model fit indices (χ^2^/df = 1.313, RMSEA = 0.012, SRMR = 0.017, CFI = 0.991, TLI = 0.990).

In this framework, the “health-information acquisition” and “health-information evaluation” dimensions capture adolescents’ general, tool-independent abilities to obtain health information from multiple sources (e.g., websites, books, and health professionals) and to judge its credibility and quality (62–64), whereas the information-acquisition behavior and information processing and comprehension dimensions of the GAI-HKA Scale focus specifically on how often and how adolescents use generative AI tools to ask health-related questions and how they subjectively process and understand AI-generated content (65). Consistent with this conceptual distinction, a supplementary CFA including all GAI-HKA and HIL items supported a model in which the two constructs were correlated but empirically separable, and the correlation between their total scores was small (r = 0.218), suggesting adequate discriminant validity.

#### 2.2.4 Self-developed generative AI health knowledge acquisition scale

In developing the GAI-HKA Scale, this study was guided by the Health Information Seeking Behavior Model and the UTAUT (26, 66). It incorporated the strategic goals of intelligent and precise development of AI-based health services outlined in China’s 14th Five-Year Plan for Digital Health. “Acquisition” in the context of the Self-Developed Generative AI Health Knowledge Acquisition Scale refers to the overall process of acquiring health-related knowledge through generative AI, which includes not only the act of seeking and searching for information but also the extent of knowledge gained and the frequency of its use (67). Three core dimensions of the scale were initially proposed: information acquisition behavior, information processing and comprehension, and knowledge application intention. Specifically, “information acquisition behavior” focuses on how users actively use generative AI to search and continue querying when facing health needs; “information processing and comprehension” emphasizes users’ evaluation of the comprehensibility, logicality, and depth of AI-generated health content; and “knowledge application intention” measures users’ motivation and confidence in transforming AI health recommendations into concrete actions and their willingness to share them.

To further refine the indicator system, grounded theory was applied, and semi-structured in-depth interviews were conducted with 30 users of different age groups. The interview themes included the use of generative AI in health inquiries, strategies for information verification, attitudes toward trusting the results, and privacy concerns. Through the three-stage analysis of open coding, axial coding, and selective coding, eight secondary indicators were summarized (e.g., “dynamic questioning ability,” “cross-source verification tendency,” “risk perception”), which were further specified into 18 tertiary conceptual items (e.g., “instruction optimization,” “comparison with authoritative sources,” “privacy trust”), thereby constructing a theory-driven initial indicator framework. This framework was then directly operationalized into the draft scale items. For instance, the concept of “dynamic questioning ability” informed items on iterative inquiry in the information acquisition behavior dimension (e.g., “Even when I cannot find satisfactory answers through web searches, I continue to ask questions of generative AI”). The concept of “comparison with authoritative sources” underlies items in the information processing and comprehension dimension, particularly those related to credibility assessment. Furthermore, concepts related to application confidence were translated into items for the knowledge application intention dimension (e.g., “When generative AI provides new health information, I am confident in trying it and applying it to my life”).

On this basis, the research team conducted two rounds of Delphi expert consultation, inviting 15 experts from the fields of medicine, informatics, health communication, sports science, and AI technology application. According to the criteria of mean importance score > 3.50, coefficient of variation (CV) < 0.25, and full-score ratio > 20%, the preliminary framework of indicators (ID) was iteratively revised and refined. Ultimately, a draft scale consisting of three primary dimensions (information acquisition behavior, information processing and comprehension, and knowledge application intention), seven secondary dimensions, and 10 items was finalized. For example, “When I have health-related questions, I will actively use generative AI for inquiry” corresponds to the “information acquisition behavior” dimension; “The health knowledge content provided by generative AI is easy for me to understand” corresponds to the “information processing and comprehension” dimension; and “I will adjust my diet or exercise habits based on the health recommendations of generative AI” corresponds to the “knowledge application intention” dimension, ensuring a high degree of alignment between the items and theoretical dimensions.

**Figure 2:**
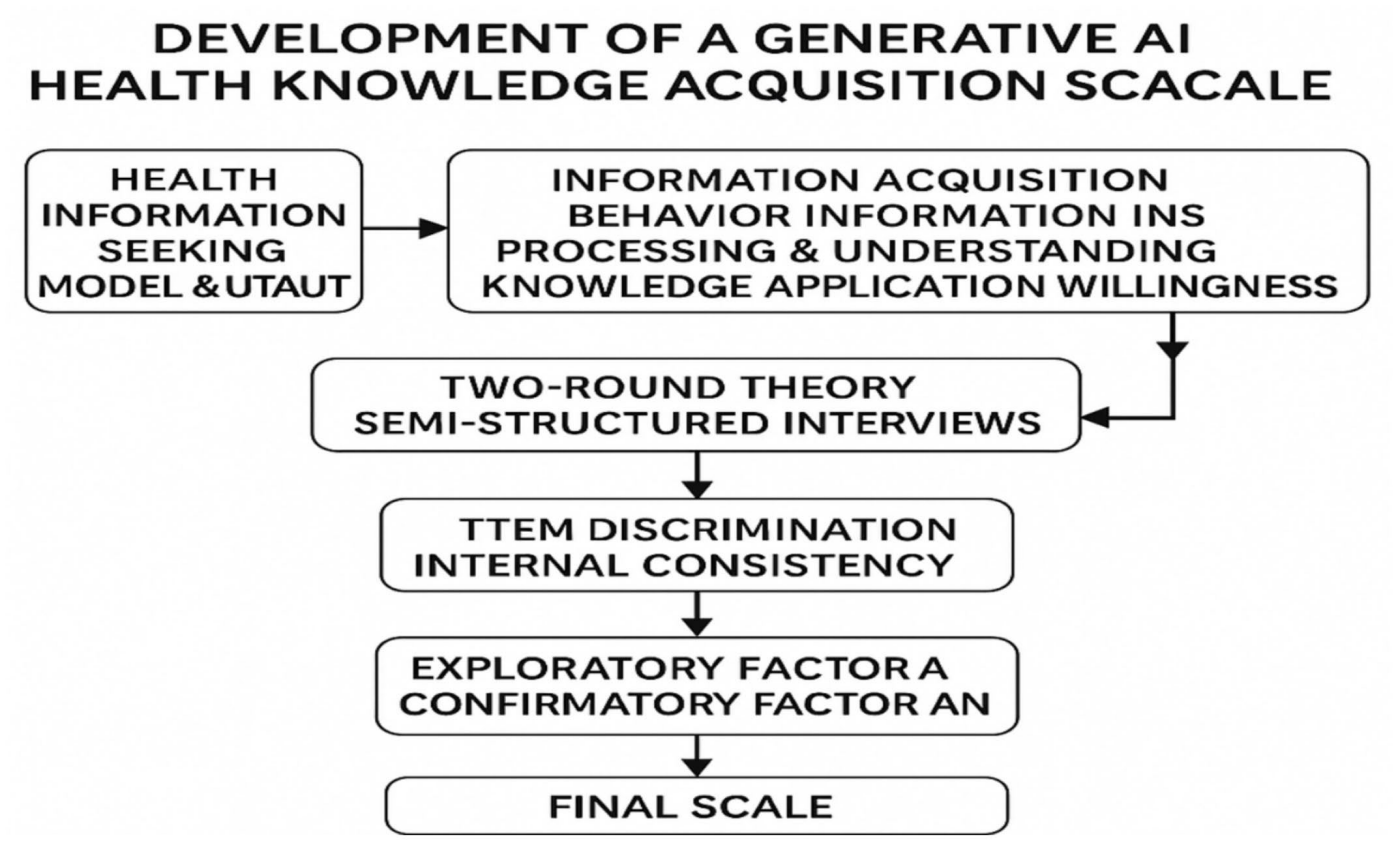
Development process of the generative AI health knowledge acquisition scale.

To test the reliability and validity of the scale, 450 valid questionnaires were collected through convenience sampling, and the following statistical analyses were conducted sequentially: (1) Item analysis: Critical ratio (CR) analysis and item–total correlation analysis were applied, and items with CR < 3.0 or correlation < 0.4 were removed; (2) Internal consistency test: The preliminary Cronbach’s *α* coefficient of the overall scale was 0.832; (3) Exploratory factor analysis (EFA): Principal component analysis with varimax rotation was used. The KMO value exceeded 0.7, Bartlett’s test was significant (*p* < 0.001), and three common factors with eigenvalues greater than 1 were extracted, explaining more than 60% of the cumulative variance. All factor loadings were greater than 0.55 with no cross-loadings. (4) CFA: A three-factor model was constructed using AMOS software. The fit indices met the criteria (χ^2^/df < 5, CFI/TLI > 0.90, RMSEA < 0.08), with all standardised loadings exceeding 0.60. In this study, χ^2^/df = 1.460, RMSEA = 0.032, SRMR = 0.042, CFI = 0.994, and TLI = 0.991, indicating good model fit and reliable construct validity. In the formal survey, the Cronbach’s *α* coefficient of the scale was 0.834. Confirmatory factor analysis further demonstrated acceptable fit indices (χ^2^/df = 1.310, RMSEA = 0.012, SRMR = 0.012, CFI = 0.999, TLI = 0.998) (Figure 2).

### 2.3 Common method deviation test

The analysis revealed that 15 factors had eigenvalues greater than 1, accounting for 57.5% of the total variance. The variance explained by the first factor was 12.0%, which did not reach the critical threshold of 40%, indicating that no significant standard method bias existed in this study.

### 2.4 Statistical analysis

SPSS 27.0 was used to perform item discrimination tests, item–total correlation analysis, and exploratory factor analysis on 450 pilot survey samples. Subsequently, confirmatory factor analysis was conducted with AMOS 26.0 to ensure the construct validity and reliability of the GAI-HKA Scale. In the formal survey, 2,306 valid questionnaires were imported into SPSS 27.0. Using the PROCESS v3.5 macro, Model 4 (5,000 bootstraps) was applied to test the mediating effect of ESE in the relationship between GAI-HKA and adolescents’ PA. Subsequently, Model 7 was used to specify a first-stage moderated mediation model in which HIL moderated the path from GAI-HKA to ESE, and the conditional indirect effects of GAI-HKA on PA through ESE were estimated at low (−1 SD), mean, and high (+1 SD) levels of HIL, along with the index of moderated mediation. In all mediation and moderation analyses, sex, age, and grade were entered as covariates.

## 3 Results

### 3.1 Descriptive statistics and correlation analysis

The results showed that the mean score of GAI-HKA was 2.89 ± 0.90 (out of 5), the mean score of PA was 28.70 ± 29.99 (out of 100), the mean score of HIL was 3.01 ± 0.74 (out of 5), and the mean score of ESE was 2.96 ± 0.83 (out of 5) (Table 2).

**Table 2:** Descriptive statistics between variables.

Variable name	Mean	SD	Min	Max
Sex	1.549	0.498	1	2
Age	14.499	1.705	12	18
Grade	2.284	1.104	1	4
GAI-HKA	2.893	0.901	1	5
PA	28.698	29.991	0	100
HIL	3.012	0.743	1	5
ESE	2.956	0.830	1	5

For the variable “SEX,” 1 = Male, 2 = Female. For the variable “AGE,” 12–18 years old. For the variable “GRADE,” 1 = Grade 7, 2 = Grade 8, 3 = Grade 10, 4 = Grade 11. All other variables are scored according to the scale. SD, standard deviation; GAI-HKA, generative AI health knowledge acquisition; PA, physical activity; HIL, health information literacy; ESE, exercise self-efficacy.

The Pearson correlation analysis of all variables is presented in Table 3. GAI-HKA showed a moderate positive correlation with PA (*r* = 0.437, *p* < 0.001), indicating that adolescents who used generative AI more frequently to acquire health knowledge demonstrated higher levels of PA. GAI-HKA was also moderately and positively correlated with ESE (*r* = 0.337, *p* < 0.001), suggesting that acquiring AI-based information helps enhance adolescents’ confidence in their exercise abilities. In addition, GAI-HKA was weakly but positively correlated with HIL (*r* = 0.218, *p* < 0.001), suggesting that adolescents with higher HIL scores were more likely to use generative AI for health knowledge acquisition. Furthermore, PA was weakly and positively correlated with HIL (*r* = 0.272, *p* < 0.001) and moderately correlated with ESE (*r* = 0.319, *p* < 0.001). HIL was also weakly and positively correlated with ESE (*r* = 0.261, *p* < 0.001), further revealing the positive interrelationships among the variables.

**Table 3:** Results of correlation analysis between variables.

Variables	GAI-HKA	PA	HIL	ESE
GAI-HKA	1			
PA	0.437***	1		
HIL	0.218***	0.272***	1	
ESE	0.337***	0.319***	0.261***	1

*p < 0.05, **p < 0.01, ***p < 0.001. The same notation applies below. GAI-HKA, generative AI health knowledge acquisition; PA, physical activity; HIL, health information literacy; ESE, exercise self-efficacy.

### 3.2 Mediating effect of exercise self-efficacy

Using SPSS 27.0 and the PROCESS 4.1 macro (Model 4), the mediating effect of ESE was tested with GAI-HKA as the independent variable, PA as the dependent variable, and ESE as the mediator. Sex, age, and grade were included as covariates in the model. The results (Table 4) showed that GAI-HKA significantly and positively predicted ESE (*β* = 0.333, *p* < 0.001), and ESE predicted PA (*β* = 0.195, *p* < 0.001). After including ESE, GAI-HKA still showed an optimistic prediction for PA (*β* = 0.376, *p* < 0.001). Bootstrap testing indicated that the indirect effect of ESE was 0.065, with a 95% confidence interval that did not contain zero, confirming that the mediating effect was statistically significant, as shown in Table 5. The effect accounted for 14.74% of the total effect.

**Table 4:** The regression analysis between variables.

Dependent variable	Independent variable	R	R^2^	F(df)	β	*t*
ESE	GAI-HKA	0.340	0.116	75.201	0.333	16.859
	Sex				0.025	0.626
	Age				−0.024	−1.098
	Grade				0.005	0.271
PA	GAI-HKA	0.476	0.226	134.489	0.376	19.190
	ESE				0.195	10.015
	Sex				0.029	0.771
	Age				0.017	1.599
	Grade				0.020	1.199

GAI-HKA, generative AI health knowledge acquisition; PA, physical activity; ESE, exercise self-efficacy.

**Table 5:** Mediating effect of exercise self-efficacy.

Effect	Effect size	Bootstrap standard error	95% Lower	95% Upper	Effect size (%)
Total	0.441	0.019	0.404	0.478	
Direct	0.376	0.020	0.337	0.414	85.26%
Indirect	0.065	0.007	0.052	0.079	14.74%

### 3.3 Moderating effect of health information literacy

SPSS 27.0 and the PROCESS 4.1 macro (Model 7) were used to examine the moderating role of HIL. Sex, age, and grade were controlled as covariates in this model. The results (Table 6) showed that GAI-HKA significantly and positively predicted ESE (*β* = 0.288, *p* < 0.001). HIL also significantly and positively predicted ESE (*β* = 0.173, *p* < 0.001). Moreover, the interaction term between GAI-HKA and HIL significantly and positively predicted ESE (*β* = 0.079, *p* < 0.001), indicating that HIL moderated the relationship between GAI-HKA and ESE.

**Table 6:** Moderating effect of health information literacy.

Dependent variable	Predictor variable	β	95% Lower	95% Upper	*t*
ESE	GAI-HKA	0.288	0.249	0.327	14.563
	HIL	0.173	0.133	0.214	8.371
	GAI-HKA × HIL	0.079	0.036	0.123	3.572
Sex (covariate)		0.032	−0.044	0.107	0.821
Age (covariate)		−0.028	−0.050	0.006	−1.461
Grade (covariate)		0.000	−0.034	0.034	−0.011

GAI-HKA, generative AI health knowledge acquisition; HIL, health information literacy; ESE, exercise self-efficacy.

**Figure 3:**
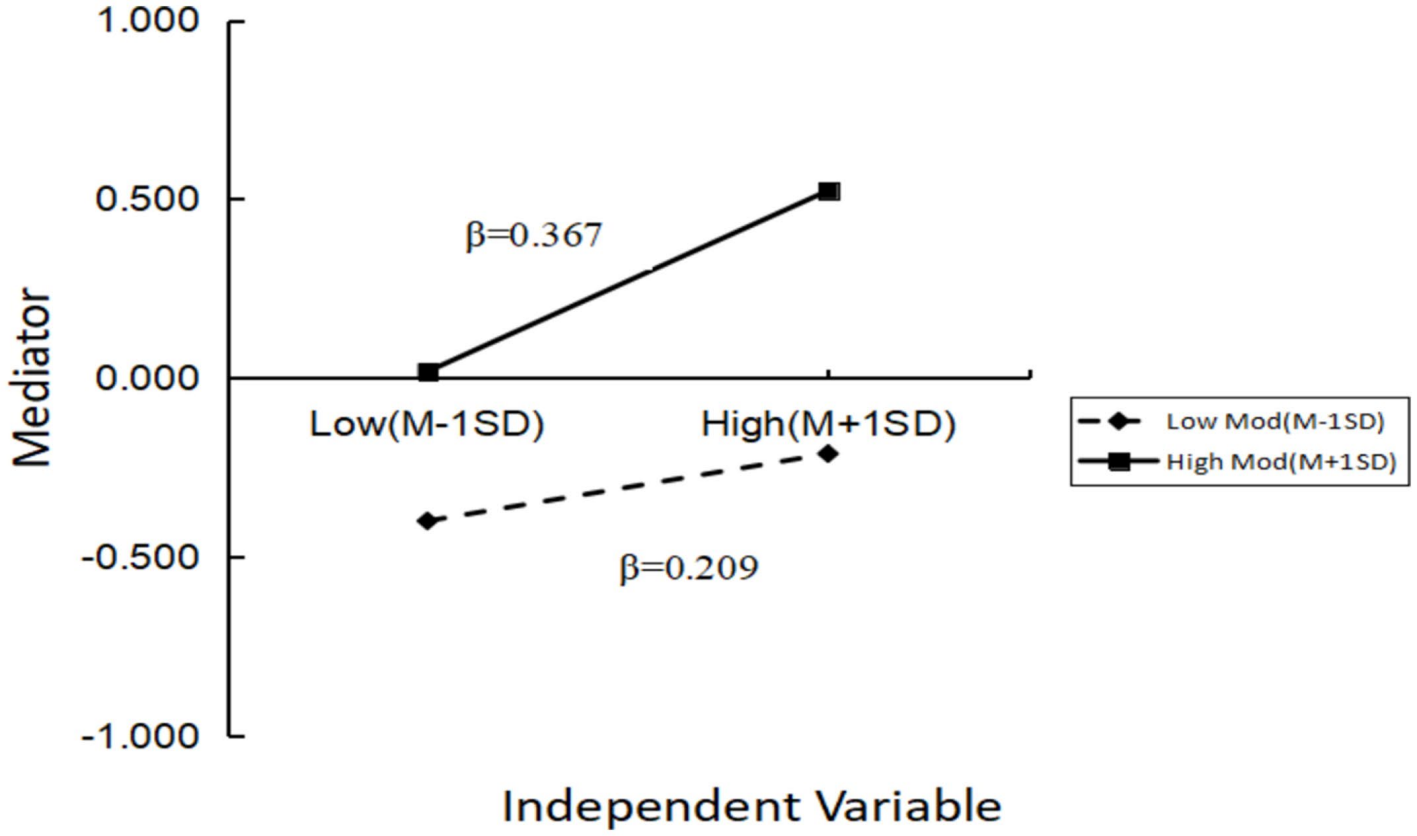
Moderating effect of health information literacy on the relationship between generative AI health knowledge acquisition and exercise self-efficacy.

To further analyze the interaction effect between GAI-HKA and HIL, the sample was divided into high and low groups based on the mean ± 1 standard deviation (SD) of HIL, and a simple slope test was conducted. As shown in Figure 3, for adolescents with high HIL, GAI-HKA significantly predicted ESE (*β* = 0.367, *p* < 0.001). For adolescents with low HIL, GAI-HKA still significantly predicted ESE, but the predictive effect was notably weaker (*β* = 0.209, *p* < 0.05). In addition, we examined whether the indirect effect of GAI-HKA on PA via ESE varied across levels of HIL. The conditional indirect effect of GAI-HKA on PA through ESE was smallest at low HIL (−1 SD; indirect effect = 0.041, 95% CI [0.029, 0.054]), intermediate at mean HIL (indirect effect 0.056=, 95% CI [0.044, 0.069]), and largest at high HIL (+1 SD; indirect effect = 0.072, 95% CI [0.055, 0.090]). The index of moderated mediation was significant (index = 0.016, 95% CI [0.007, 0.025]), indicating that the mediating role of ESE in the association between GAI-HKA and PA was stronger among adolescents with higher HIL, consistent with the hypothetical model shown in Figure 1.

## 4 Discussion

The shaping of adolescents’ health behaviors is closely related to lifelong development, and their level of PA is an essential manifestation of health literacy (68). Focusing on GAI as an emerging technology, this study is the first to reveal the dual pathways through which Chinese adolescents acquire health knowledge via AI to influence their PA behaviors. The direct effect was significant, indicating that the instant accessibility of AI-based health information directly stimulates exercise engagement. At the same time, ESE served as a key mediator, confirming that AI indirectly drives behavior by strengthening adolescents’ confidence in their exercise capabilities. Furthermore, HIL effectively moderated this process, highlighting the boundary role of individual information-processing ability in shaping the utility of technology. Together, these findings provide a precise theoretical framework of “technological empowerment–psychological mechanism–behavioral transformation” for AI-based health interventions.

### 4.1 The impact of generative AI health knowledge acquisition on adolescents’ physical activity

The data analysis results of this study showed that GAI-HKA had a significant positive predictive effect on adolescents’ PA, thus supporting Hypothesis H1. This finding is highly consistent with the longitudinal conclusions of previous research on the relationship between online health information seeking and PA (69). This is further corroborated by the positive association observed in a cross-sectional study (70). According to the UTAUT, the high “performance expectancy” of generative AI leads adolescents to believe that its exercise recommendations are more targeted and effective. For instance, a teenager may ask for a workout plan tailored to their fitness goals or inquire about specific exercises for improving a sport, which could enhance their motivation to engage in physical activity (26). In contrast, its low “effort expectancy” reduces the costs of information retrieval and comprehension. Together, these factors enhance “usage intention,” which, under the influence of “facilitating conditions” (e.g., campus Wi-Fi, widespread mobile device availability), translates digital behaviors into offline exercise practices (71). This suggests that GAI can serve as a powerful tool in promoting adolescents’ physical activity, especially when personalized recommendations are easily accessible.

In the Chinese context, the “Double Reduction” policy, implemented to reduce homework and after-school tutoring, has freed up more discretionary after-school time for students. This policy aims to decrease academic pressure and increase students’ free time, enabling them to engage more in extracurricular activities and self-directed learning. As a result, this policy creates more opportunities for students to explore educational tools, such as generative AI, which can be used to enhance their physical activity (72). Generative AI fills the information gap between traditional physical education classes and family guidance: after school (73), students can use smartphones or tablets to instantly ask questions such as “How can I complete a 15-min core workout in the dormitory?” or “What techniques can improve my basketball shooting accuracy (74)?” AI responds with personalised solutions through multimodal outputs, such as text, images, and voice interaction (75). This approach not only aligns with the Ministry of Education’s push for “smart sports” in teaching reform but also meets parents’ urgent expectations for safe and scientific exercise (76). In addition, algorithmic recommendations and peer-sharing features further amplify the demonstration effect, making “AI-assisted training” a new topic of campus socialization (77). Under the combined influence of institutional, technological, and cultural support, where cultural support is characterized by the increasing emphasis on health and wellness within Chinese educational and social systems, as well as the growing acceptance of technology in daily life, GAI-HKA thus exerts a significantly enhanced positive impact on adolescents’ PA (78–80).

### 4.2 The mediating role of exercise self-efficacy in the relationship between generative AI health knowledge acquisition and adolescents’ physical activity

The results of the structural equation model indicated that ESE played a partial mediating role in the relationship between GAI-HKA and adolescents’ PA, thereby supporting Hypothesis H2. This finding is consistent with the conclusions of previous research regarding the chain effect of “online information → self-efficacy → PA,” and also resonates with the longitudinal observations reported in subsequent studies (81). Social cognitive theory emphasizes that external environments shape individuals’ efficacy beliefs through mechanisms of “modeling–feedback (82).” In this study, generative AI, by providing exercise recommendations in an instant, personalized, and continuously positive interactive manner, effectively served as a credible “virtual coach (83).” Through the repeated successful execution of AI-recommended activities, reinforced by algorithmic affirmation and peer recognition, adolescents gradually developed the subjective judgment that “I can complete and sustain exercise,” reflecting enhanced ESE (84).

According to the core propositions of self-efficacy theory, strengthened efficacy beliefs directly drive the initiation, intensity, and persistence of behavior. Adolescents are more willing to set higher exercise goals, devote greater time and effort, and remain resilient when facing challenges such as academic demands or unfavorable weather conditions (43). Large-sample evidence from previous research further demonstrated that a one standard deviation increase in ESE significantly raised the probability of meeting the daily moderate-to-vigorous PA guidelines (85).

Therefore, GAI-HKA does not operate through a simple “information push → behavior execution” pathway. Instead, by enhancing ESE as a psychological hub, it transforms digital information into sustained offline exercise behaviors, forming a complete psychological mechanism of “information input → efficacy reinforcement → behavioral output (86).”

### 4.3 The moderating role of health information literacy in the relationship between generative AI health knowledge acquisition and adolescents’ physical activity

The results of this study demonstrated that HIL significantly moderated the relationship between GAI-HKA and adolescents’ PA, thus supporting Hypothesis H3. Further analysis, based on M ± 1 SD, divided the sample into high and low HIL groups. The simple slope tests revealed that both slopes were significantly positive, although their magnitudes differed, showing a typical pattern: “the higher, the steeper; the lower, still rising.” Specifically, for adolescents with high HIL, exercise recommendations provided by generative AI can be rapidly identified, compared, and integrated (22). Students engaged in deep processing of information via the “central route” (as explained by the elaboration likelihood model, ELM), transforming each AI interaction into a new training plan or self-challenge (51). As the frequency of AI use increased, their PA levels rose sharply, with the slope significantly greater than that of the low HIL group (87, 88).

In contrast, for adolescents with low HIL, although their abilities in information screening and judgment were relatively weak (89), the conversational guidance and instant error-correction features of AI lowered the threshold for use (90). These students relied more on the “peripheral route” to gain emotional support and behavioral modelling, and their PA also increased significantly with higher AI use. However, the slope was relatively flat (91). In other words, high HIL functions as an “amplifier,” magnifying the effect of GAI-HKA on PA several fold, while low HIL serves as a “booster,” exerting a weaker force but still ensuring that the positive impact is preserved rather than negated.

From a practical perspective, these findings suggest several actionable strategies for promoting physical exercise and health among adolescents. First, schools and families can purposefully integrate generative AI tools into health and physical education, using them to provide adolescents with accessible, low-cost, and personalized exercise guidance (e.g., individualized training plans, real-time adjustment of workloads, and tailored advice for overcoming barriers to activity) instead of relying solely on generic recommendations (92). Second, because exercise self-efficacy emerged as a key psychological mechanism, intervention programs should not only deliver information but also deliberately structure AI-assisted activities to enhance adolescents’ confidence—for example, by encouraging graded goal-setting, providing timely feedback on progress, and using AI as a “virtual coach” that reinforces successful experiences (93, 94). Third, the moderating role of health information literacy indicates that efforts to promote AI-based exercise interventions need to be accompanied by systematic training in how to search for, evaluate, and apply health information, so that adolescents can distinguish reliable AI-generated content from misleading or unsafe suggestions (95). Finally, policymakers and platform designers should consider developing age-appropriate guidelines and safeguards for the use of generative AI in youth health contexts, thereby creating supportive digital environments in which adolescents can safely translate AI-derived health knowledge into sustained physical activity behaviors (96).

## 5 Conclusion

This study confirmed that GAI-HKA significantly promotes adolescents’ PA, and this effect is reinforced through ESE. HIL further moderated the relationship at both high and low levels, showing a pattern of “the higher, the steeper; the lower, still rising.” These findings suggest that when AI-generated health advice aligns with adolescents’ existing efficacy beliefs and is further supported by their ability to evaluate and apply information, the combined effect catalyzes sustained growth in PA participation. Therefore, in promoting AI-based fitness tools, schools and families should simultaneously strengthen students’ training in information literacy and cultivate efficacy beliefs to maximize the long-term benefits of digital health interventions.

## 6 Limitations and strengths

This study has several strengths. It is the first to integrate generative AI health knowledge acquisition (GAI-HKA), exercise self-efficacy (ESE), and health information literacy (HIL) into a chain model of adolescents’ physical activity (PA). The sample size was sufficient, the measurement tools were localized (97), and both reliability and validity were satisfactory (98), providing a new theoretical perspective and empirical evidence for digital health interventions (99, 100).

Nevertheless, some limitations should be acknowledged. First, the cross-sectional design limits causal inference, and future studies should adopt longitudinal or experimental designs for validation. Second, all data were based on self-reported questionnaires, which may be subject to social desirability or recall bias; subsequent research could incorporate objective measures such as wearable devices or behavioral logs. Third, the sample was limited to southern Chinese cities, where cultural and economic contexts are relatively homogeneous, so caution is warranted when generalizing the findings. Fourth, potential confounding variables, such as parental support and school sports environments, were not included, which may have led to an underestimation or overestimation of the actual effects.

## Data Availability

The original contributions presented in the study are included in the article/supplementary material, further inquiries can be directed to the corresponding authors.
