# Supplementary material for: The impact of generative AI health knowledge acquisition on adolescents’ physical activity: the mediating role of exercise self-efficacy and the moderating effect of health information literacy
Source: Front Public Health. 2026 Jan 8;13:1705265. doi: 10.3389/fpubh.2025.1705265 (PMC12823970; doi:10.3389/fpubh.2025.1705265)
Supplement: Supplementary file 1 [file Supplementary_file_1.docx]

Supplementary Material

# Supplementary Figures and Tables

## Supplementary Tables

Item analysis: We performed critical-ratio (CR) analysis and item–total correlation analysis, and removed items with CR < 3.0 or item–total correlation < 0.40. The results are presented in Table 1.

**Table 1.** Item analysis for the Generative AI Health Knowledge Acquisition scale.

| Dimension | items | t | r |
| --- | --- | --- | --- |
| information acquisition behavior | I proactively consult generative AI (e.g., ChatGPT, intelligent assistants) when I have health-related questions. | -15.586*** | 0.661*** |
|  | I frequently use generative AI to look for new information about healthy diet or physical activity. | -13.926*** | 0.618*** |
|  | In my daily life, I tend to use generative AI as my preferred source for obtaining health information. | -15.641*** | 0.657*** |
|  | Even when I cannot find satisfactory answers through web searches, I continue to ask questions of generative AI. | -15.751*** | 0.670*** |
| information processing and comprehension | I find the health information provided by generative AI easy to understand. | -13.226*** | 0.605*** |
|  | The health recommendations generated by generative AI are well organized and logically coherent. | -12.041*** | 0.579*** |
|  | I believe generative AI can provide more in-depth explanations of health topics than traditional search engines. | -12.297*** | 0.567*** |
| knowledge application intention | In my daily life, I adjust my diet or exercise habits based on health advice from generative AI. | -15.527*** | 0.639*** |
|  | When generative AI provides new health information, I am confident in trying it and applying it to my life. | -17.964*** | 0.673*** |
|  | I am willing to proactively share health knowledge obtained from generative AI with family or friends. | -14.259*** | 0.633*** |

Note: **p*< 0.05, ***p* < 0.01, ****p*< 0.001. The same notation applies below.

Internal consistency: The overall Cronbach’s a for the scale was 0.832. Cronbach’s a for each dimension are shown in Table 2.

**Table 2.** Summary of reliability analysis.

|  | number of terms | Cronbach’s a |
| --- | --- | --- |
| Information-seeking behaviors | 4 | 0.863 |
| Information processing and comprehension | 3 | 0.850 |
| knowledge application intention | 3 | 0.889 |
| Overall | 10 | 0.832 |

Exploratory Factor Analysis (EFA): Principal component analysis with varimax rotation was conducted. The KMO value exceeded 0.70, and Bartlett’s test of sphericity was significant (*p*< 0.001). Three common factors with eigenvalues greater than 1 were extracted, accounting for a cumulative variance of over 60%. All items had factor loadings above 0.55 with no cross-loadings. The results are shown in Table 4.

**Table 3.** KMO and Bartlett’s Test

| Kaiser-Meyer-Olkin Measure of Sampling Adequacy. |  | 0.816 |
| --- | --- | --- |
| Bartlett's Test of Sphericity | Approx. Chi-Square | 2288.426 |
|  | df | 45 |
|  | Sig. | 0.000 |

**Table 4.** Rotated component matrix from exploratory factor analysis

| **Item** | **Factor** | | |
| --- | --- | --- | --- |
|  | **1** | **2** | **3** |
| AI1 | 0.823 |  |  |
| AI2 | 0.813 |  |  |
| AI3 | 0.865 |  |  |
| AI4 | 0.810 |  |  |
| AI5 |  |  | 0.860 |
| AI6 |  |  | 0.858 |
| AI7 |  |  | 0.868 |
| AI8 |  | 0.887 |  |
| AI9 |  | 0.873 |  |
| AI10 |  | 0.895 |  |

Extraction Method: Principal Component Analysis.

Rotation Method: Varimax with Kaiser Normalization.

a Rotation converged in 5 iterations.

## Supplementary Figures

Confirmatory Factor Analysis (CFA): A three-factor model was constructed using AMOS (Figure 1). The fit indices ($\chi$^2^/df = 1.460, RMSEA = 0.032, SRMR = 0.042, CFI = 0.994, TLI = 0.991) indicated good model fit and reliable structural validity.


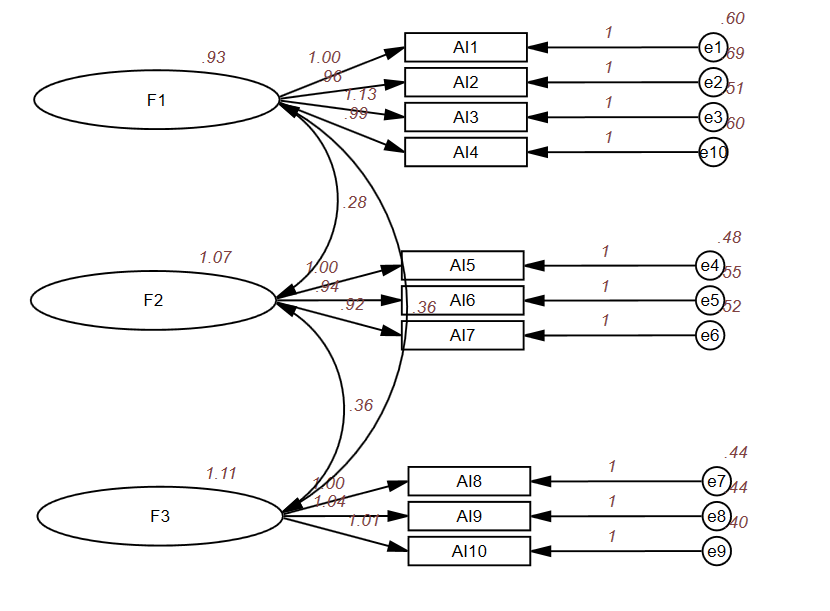


**Figure 1.** Confirmatory factor analysis model.
